# Association between participation self-efficacy and participation in stroke survivors

**DOI:** 10.1186/s12883-022-02883-z

**Published:** 2022-09-22

**Authors:** Suzanne H S Lo, Janita P C Chau, Simon K Y Lam, Ravneet Saran, Kai Chow Choi, Jie Zhao, David R. Thompson

**Affiliations:** 1 Nethersole School of Nursing, Faculty of Medicine, Chinese University of Hong Kong, Hong Kong SAR, China; 2 School of Nursing and Midwifery, Queen’s University Belfast, Belfast, UK

**Keywords:** Social participation, Self-efficacy, Stroke, Stroke rehabilitation

## Abstract

**Background:**

Most stroke survivors face restrictions in functional disability and social participation, which can impede their recovery and community reintegration. Participation self-efficacy refers to survivors’ confidence in using strategies to manage participation in areas including community living and work engagement. This study aimed to assess the association between participation self-efficacy and participation among stroke survivors.

**Methods:**

This study adopted a cross-sectional correlational design with a convenience sample of 336 stroke survivors recruited from five hospitals in China. Participation self-efficacy was measured using the Chinese version of the Participation Strategies Self-Efficacy Scale (PS-SES-C) and participation measured using the Chinese version of the Reintegration to Normal Living Index (RNLI-C). The association between participation self-efficacy and participation was examined using multiple regression analysis with adjustment for potential confounders.

**Results:**

Participants had a mean age of 69.9 ± 11.5 years, with most (81.6%) having an ischaemic stroke, and more than half (61.6%) a first-ever stroke. After adjustment for potential confounders, every 10-point increase in the PS-SES-C total score was significantly associated with an average 1.3-point increase in the RNLI-C total score (B = 1.313, SE = 0.196, *p* < 0.001).

**Conclusions:**

This study demonstrates that participation self-efficacy is significantly associated with participation among Chinese community-dwelling survivors of a mild or moderate stroke. This suggests that rehabilitation programmes for stroke survivors may be more effective if they incorporate participation-focused strategies designed to enhance self-efficacy.

(229 words).

**Supplementary Information:**

The online version contains supplementary material available at 10.1186/s12883-022-02883-z.

## Background

Stroke is a global public health problem that imposes a significant human and economic burden. It is expected that by the year 2050 that there will be 200 million stroke survivors globally, with an additional 30 million new stroke cases and 12 million deaths every year [1]. Besides its evident impact on mortality, stroke also results in diverse emotional, physical, cognitive, and social problems in survivors [2].

Participation is defined as the ability to be involved in life situations, while social participation refers specifically to one’s involvement in activities consisting of interpersonal interactions in society or the community [3]. Participation is a multi-faceted and complex process that intersects the person, task, and environment, requiring survivors to strategise between competing personal and environmental factors to achieve desired tasks [4]. A longitudinal study of 1, 294 middle-aged and older stroke survivors in China revealed that more than half of them did not engage in any social participation after stroke [5]. A review of 70 studies comprising 4,816 stroke survivors reported that while survivors’ contact with their families stayed relatively stable after stroke, survivors significantly reduced contact with friends and participation in social activities [6]. In addition, a recent review of 81 studies comprising 11,815 stroke survivors found depressive symptoms, cognitive functioning, mobility, and activity limitations had the strongest correlations with participation [7]. Studies have also highlighted the detrimental impact of impaired balance function and independence level after stroke on the social participation of stroke survivors [8].

Given that participation is a vital outcome in stroke recovery and the reintegration of stroke survivors into the community, participation-focused rehabilitation interventions are essential [9]. Studies have revealed an increased risk of recurrent stroke by up to 64%, in socially isolated people, as well as the association of inadequate social support and community participation with post-stroke depression [10, 11]. Enhancing survivors’ participation levels has been suggested to improve their post-stroke quality of life and promote their independence, autonomy, and social inclusion [12, 13]. However, current rehabilitation interventions continue to focus largely on survivors’ physical recovery and insufficiently on participation and community reintegration despite the prevalence of persistent participation restrictions that can extend into the chronic phase of stroke [14]. Facilitators of participation include the physical environment, accessibility to required services, personal perseverance, adaptability, ability to manage emotional challenges, and social support [15–17]. It is crucial to understand the relationship between facilitating factors and participation to promote stroke survivor’s reintegration into society.

Recent studies have emphasised the critical role of self-efficacy in stroke rehabilitation [18, 19]. Self-efficacy is defined as a person’s belief in their own capabilities to plan and perform actions to reach their goals [20]. Stroke survivors with stronger self-efficacy are expected to be more initiating and persistent in the performance of important activities to achieve their goals during the recovery process [21]. A systematic review of 17 studies noted that self-efficacy was associated with positive outcomes, including improvements in mobility, activities of daily living, and health-related quality of life, and a reduction in depressive symptoms [22]. A prospective study of 52 stroke survivors further reported the role of balance self-efficacy and fall self-efficacy in predicting community reintegration [23]. A Chinese study also indicated that aside from physical function, self-efficacy was correlated most strongly with participation [24].

However, while studies have examined the relationship between self-efficacy and participation, most research has largely focused on self-efficacy in relation to survivors’ balance or concerns about falling, or general stroke management. No study has specifically considered the role of survivor’s participation self-efficacy, which constitutes survivors’ confidence in using strategies to manage participation in multiple areas, including community living and work engagement [25]. An exploration of the association between participation self-efficacy and participation after stroke may inform the development of participation-focused interventions that help survivors foster effective strategies and promote positive recovery outcomes.

## Methods

### Aim

The aim of this study was to examine the association between participation self-efficacy and participation.

### Design

This study adopted a cross-sectional correlational design and was conducted in the neurology departments of five hospitals in Kunming, China. STROBE cross-sectional reporting guidelines were followed [26].

### Participants

Stroke survivors who met the following criteria were invited to participate: (1) aged 18 years or above, (2) clinically diagnosed with a first or recurrent ischaemic or haemorrhagic stroke, (3) had mild or moderate stroke (National Institutes of Health Stroke Scale [NIHSS] < 16), (4) resided in a community-based setting, (5) had completed initial acute/rehabilitation care, and (6) able to provide consent to participate. Survivors with (1) unstable medical conditions, (2) cognitive impairment (a Montreal Cognitive Assessment [MoCA] score ≤ 2nd percentile), and (3) severe aphasia were excluded.

This study was a secondary data analysis of a study that aimed to examine the psychometric properties of the Chinese version of the Participation Strategies Self-Efficacy Scale (PS-SES-C) [27]. The previous study recruited a total of 336 participants [27], thereby enabling us to detect an explanatory variable for participation with an effect size as small as R2 = 0.023 and 80% power at 2-sided 5% level of significance using linear regression. This means that the explanatory variable of participation self-efficacy, which explains at least 2.3% of the variance of participation, would be detectable with 80% power at 5% level of significance. The sample size was thus adequate for the purpose of examining the association between participation self-efficacy and participation. Power analysis was performed using PASS 16.0 (NCSS, LLC. Kaysville, Utah, USA).

### Data collection

Ethical approval was obtained from the Survey and Behavioural Research Ethics Committee of the Chinese University of Hong Kong (Reference no.: SBRE-19–412). Research assistants, who were undergraduate nursing students and had received training in administering questionnaires, first screened medical records from the participating neurology departments to identify eligible participants. They approached eligible survivors and explained the study to them in detail. The study conformed to the principles of the Declaration of Helsinki, and participants were informed of their rights to participate voluntarily and withdraw from the study at any time without influence on their present or future care. Their anonymity and confidentiality were also assured. After providing written informed consent, participants completed a demographic sheet and a set of self-reported questionnaires. Their clinical information was collected by reviewing their medical records.

### Measurements

#### Participation self-efficacy

Participation self-efficacy was measured using the Chinese version of the Participation Strategies Self-Efficacy Scale (PS-SES), which consists of 35 items evaluating six dimensions of participation self-efficacy, namely managing home participation, staying organised, planning and managing community participation, managing work/productivity, managing communication, and advocating for resources [25, 27]. Subjective responses are rated on an analogue scale of 1 to 10. The total score ranges from 35 to 350. A higher score indicates a higher level of participation self-efficacy. The PS-SES has shown satisfactory internal consistency (Cronbach’s α for all domains = 0.86–0.93) [25]. In this study, Cronbach’s α of the six domains of the Chinese version of the PS-SES (PS-SES-C) ranged from 0.90 to 0.95 [27].

#### Participation

Participation was measured using the Chinese version of the Reintegration to Normal Living Index (RNLI), which includes 11 items assessing the degree by which a person with trauma or a neurological disease has reintegrated into a normal life [28]. The RNLI covers areas of participation specific to social activities and community involvement, including social activities, role within the family, comfort with relationships, and ability to handle life events. Each item is rated on an 11-point rating scale from 0 (least agreement) to 10 (greatest agreement). The summation score (0 – 110) is converted to a range between 0 to 100 by dividing by 1.1 [28]. Higher scores represent a greater extent of participation. The Cronbach’s α of the Chinese version of RNLI (RNLI-C) among the Chinese population was 0.92 [29].

#### Mobility

Mobility was measured using the Chinese version of the Rivermead Mobility Index (RMI), which consists of 15 items focusing on functional mobility in gait, balance, and transfers [30]. Each item is scored on a 2-point scale (0 = “no”, 1 = “yes”). The assessors (trained nursing students) scored item five by direct observation. The total score varies from 0 to 15, with higher scores indicating better mobility. The Chinese version of the RMI (RMI-C) was shown to be valid and sensitive (coefficients of reproducibility > 0.9, coefficients of scalability > 0.7) among the Chinese population [31].

#### Performance in activities of daily living (ADLs)

ADLs performance was measured using the Chinese version of the Modified Barthel Index (MBI), which consists of 15 items rated on a 5-point scale, with the total score ranging from 0 to 100 [32]. A higher score indicates a higher level of performance in ADLs. The inter- and intra-rater reliability of the Chinese version of the MBI (MBI-C) was good [33].

### Demographic and clinical information

Demographic and clinical information, including age, sex, educational level, marital status, pre-morbid employment and financial role in family, current and past medical history, use of assistive aids, type of stroke, lesion side, stroke frequency, severity of stroke graded by the NIHSS (range 0–42) [34], and cognitive status (measured by MoCA) [35], were recorded.

### Data analysis

Normality of continuous data was assessed on the basis of skewness statistics and normal probability plots. Data were presented using appropriate descriptive statistics. The outcome of interest was participation as assessed by the total score of the RNLI-C. Bivariate analysis between the RNLI-C total score and each of the demographic and clinical characteristics, participation self-efficacy (PS-SES-C), mobility (RMI-C), and performance in ADLs (MBI-C) was conducted using Pearson correlation coefficients, independent t-tests, or one-way ANOVA, as appropriate. Association between participation self-efficacy (PS-SES-C) and participation (RNLI-C) was examined using multiple regressions in a hierarchical fashion for successively adjusting participants’ demographic and clinical characteristics, mobility, and ADLs performance, which showed significant association with participation in bivariate analyses. All statistical analyses were performed using IBM SPSS 26.0 (IBM Crop, Armonk, NY). All statistical tests were 2-sided with the level of significance set at 0.05.

## Results

### Sample characteristics

The mean age of the 336 participants was 69.9 ± 11.5 years, and 53.0% of them were men. Most (81.6%) suffered from ischaemic stroke, and 61.6% had a first stroke. Nearly a third (31.8%) used assistive aids. Over two-thirds (70.5%) had hypertension, over a quarter (26.8%) diabetes, and nearly a fifth (17.3%) heart disease. The participants had an average RNLI-C total score of 58.7 ± 22.9 (over a possible range of 0 to 100) and PS-SES-C total score of 228.0 ± 61.0 (over a possible range of 35 to 350). Table 1 presents participants’ demographic and clinical characteristics.

**Table 1:** Demographic and clinical characteristics of the study sample (*N* = 336)

	Mean (SD)/n (%)
**Demographic characteristics**	
Age (years) †	69.9 (11.5)
Sex	
Male	178 (53.0%)
Female	158 (47.0%)
Educational level	
No formal education / primary	85 (27.9%)
Secondary	144 (47.2%)
Post-secondary or above	76 (24.9%)
Marital status	
Married	262 (78.4%)
Divorced / widowed	72 (21.6%)
Pre-morbid employment	
Full-time	225 (71.7%)
Part-time	22 (7.0%)
Retired	67 (21.3%)
Pre-morbid financial role in family	
Major	68 (20.3%)
Shared	197 (58.8%)
No role	70 (20.9%)
**Clinical characteristics**	
History of hypertension	
No	99 (29.5%)
Yes	237 (70.5%)
History of diabetes	
No	246 (73.2%)
Yes	90 (26.8%)
History of heart disease	
No	278 (82.7%)
Yes	58 (17.3%)
Assistive aids used	
None	221 (68.2%)
Crutch and / or wheelchair	103 (31.8%)
Type of stroke	
Ischaemic stroke	270 (81.6%)
Haemorrhagic/both ischaemic and haemorrhagic stroke	61 (18.4%)
Lesion side	
Left	117 (35.5%)
Right	150 (45.5%)
Both	63 (19.0%)
Stroke frequency	
One	204 (61.6%)
Two or more	127 (38.4%)
Stroke symptom severity	
Mild (NIHSS score: 0–4)	245 (73.6%)
Moderate (NIHSS score: 5–15)	88 (26.4%)
MoCA total score †	18.9 (7.4)
MBI-C total score †	84.9 (21.3)
RMI-C total score †	10.4 (5.1)

Data marked with † are presented as mean (standard deviation), all others are presented as frequency (%)

*NIHSS* National Institutes of Health Stroke scale, *MoCA* Chinese version of the Montreal Cognitive Assessment, *MBI-C* Chinese version of the Modified Barthel Index, *RMI-C* Chinese version of the Rivermead Mobility Index, *SD* Standard deviation

### Characteristics associated with participation

The bivariate analysis showed educational level, marital status, pre-morbid financial role in family, history of diabetes, history of heart disease, use of assistive aids, type of stroke, lesion side, stroke frequency, and stroke symptom severity were significantly associated with participation (all *p* < 0.05) (Table 2). Stroke survivors with better ADLs performance, a higher level of mobility, and higher participation self-efficacy were likely to have better participation (all *p* < 0.05) (Table 2).

**Table 2:** Bivariate analyses between participation and characteristics, ADLs performance, mobility, and participation self-efficacy

	Mean (SD)/ Correlation with RNLI-C total score	*p*-value
**Demographic characteristics**		
Age (years)	-0.094	0.085
Sex		
Male	57.2 (23.8)	0.220
Female	60.3 (21.9)	
Educational level		
No formal education / primary	54.5 (21.1)	< 0.001
Secondary	55.4 (24.0)	
Post-secondary or above	69.8 (20.4)	
Marital status		
Married	61.1 (21.3)	< 0.001
Divorced / widowed	49.8 (26.4)	
Pre-morbid employment		
Full-time	58.9 (24.2)	0.078
Part-time	50.0 (19.9)	
Retired	62.7 (18.7)	
Pre-morbid financial role in family		
Major	56.8 (23.0)	0.009
Shared	61.7 (23.2)	
No role	52.2 (21.0)	
**Clinical characteristics**		
History of hypertension		
No	59.5 (24.3)	0.676
Yes	58.4 (22.4)	
History of diabetes		
No	60.3 (21.5)	0.031
Yes	54.2 (26.0)	
History of heart disease		
No	60.3 (22.4)	0.006
Yes	51.2 (24.1)	
Assistive aids used		
None	65.7 (18.7)	< 0.001
Crutch and / or wheelchair	42.6 (23.6)	
Type of stroke		
Ischaemic stroke	62.1 (21.8)	< 0.001
Haemorrhagic / both Ischaemic and haemorrhagic stroke	45.1 (22.3)	
Lesion side		
Left	51.7 (23.1)	< 0.001
Right	59.4 (22.5)	
Both	67.4 (19.7)	
Stroke frequency		
One	53.7 (22.8)	< 0.001
Two or more	66.6 (21.1)	
Stroke symptom severity		
Mild (NIHSS score: 0–4)	66.5 (18.7)	< 0.001
Moderate (NIHSS score: 5–15)	37.7 (20.4)	
**ADLs performance, mobility & participation self-efficacy**		
MBI-C total score	0.671	< 0.001
RMI-C total score	0.741	< 0.001
PS-SES-C total score	0.699	< 0.001

*RNLI-C* Chinese version of Reintegration to Normal Living Index, *NIHSS* National Institutes of Health Stroke Scale, *MBI-C* Chinese version of the Modified Barthel Index, *RMI-C* Chinese version of the Rivermead Mobility Index, *PS-SES-C* Chinese version of the Participation Strategies Self-Efficacy Scale, *ADLs* Activities of Daily Living, *SD* Standard deviation

### Association between participation self-efficacy and participation

Multiple regression analyses conducted in a hierarchical fashion were used to evaluate the association between participation self-efficacy and participation in stroke survivors. The crude unadjusted Model 1 (Table 3) indicated that every 10-point increase in the PS-SES-C total score was significantly associated with an average increment of 2.628 points in the RNLI-C total score (B = 2.628, SE = 0.147, *p* < 0.001). After successively adjusting for the potential confounders identified in bivariate analyses, namely the demographic and clinical characteristics, mobility, and ADLs performance, which showed a significant association with the RNLI-C total score, the association between the PS-SES-C total score and the RNLI-C total score remained significant (Models 2 to 4, Table 3). The fully adjusted Model 4 indicated that every 10-point increase in the PS-SES-C total score was significantly associated with an average of 1.313-point increase in the RNLI-C total score (B = 1.313, SE = 0.196, *p* < 0.001) (Model 4, Table 3).

**Table 3:** Association between participation self-efficacy and participation

	**Model 1**			**Model 2**			**Model 3**			**Model 4**		
**Factors**	B	SE	*p*-value	B	SE	*p*-value	B	SE	*p*-value	B	SE	*p*-value
PS-SES-C total score a	2.628	0.147	< 0.001	2.639	0.146	< 0.001	1.997	0.193	< 0.001	1.313	0.196	< 0.001
**Demographic characteristics**												
Educational level												
No formal education / primary (ref)												
Secondary				2.476	2.263	0.275	0.664	2.264	0.770	-0.257	2.076	0.902
Post-secondary or above				7.111	2.652	0.008	4.554	2.661	0.088	4.162	2.432	0.088
Marital status												
Married (ref)												
Divorced / widowed				-3.665	2.299	0.112	-2.572	2.369	0.279	-0.871	2.160	0.687
Pre-morbid financial role in family												
Major (ref)												
Shared				10.394	2.314	< 0.001	6.805	2.367	0.004	6.818	2.153	0.002
No role				0.052	2.983	0.986	-2.508	2.940	0.394	-3.562	2.676	0.184
**Clinical characteristics**												
History of diabetes												
No (ref)												
Yes							-2.663	2.037	0.192	-1.993	1.849	0.282
History of heart disease												
No (ref)												
Yes							-0.432	2.444	0.860	-0.435	2.216	0.844
Assistive aids used												
None (ref)												
Crutch and / or wheelchair							-7.261	2.199	0.001	0.070	2.235	0.975
Type of stroke												
Ischaemic stroke (ref)												
Haemorrhagic / both ischaemic and haemorrhagic stroke							0.154	2.478	0.951	2.149	2.263	0.343
Lesion side												
Left (ref)												
Right							1.711	1.956	0.383	1.736	1.773	0.329
Both							4.092	2.839	0.151	4.183	2.579	0.106
Stroke frequency												
One (ref)												
Two or more							7.168	2.033	< 0.001	5.717	1.853	0.002
Stroke symptom severity												
Mild (NIHSS score: 0–4)												
Moderate (NIHSS score: 5–15)							-6.561	2.609	0.013	-1.966	2.480	0.429
**ADLs performance and functional mobility**												
MBI total score										0.064	0.067	0.341
RMI-C total score										1.813	0.312	< 0.001

^a^ The regression coefficient corresponds to every 10-point increase in PS-SES-C total score

Model 1: unadjusted crude model

Model 2: with adjustment for the socio-demographic characteristics which showed significant association with RNLI-C total score in univariate analysis

Model 3: with adjustment for the clinical characteristics which showed significant association with RNLI-C total score in univariate analysis + covariates in Model 2

Model 4: with adjustment for ADLs performance and functional mobility + covariates in Model 3

Ref, reference category of categorical independent variable, *NIHSS* National Institutes of Health Stroke Scale, *MBI* Modified Barthel Index, *RMI-C* Chinese version of the Rivermead Mobility Index, *B* Regression coefficient, *SE* Standard error, *PS-SES-C* Chinese version of the Participation Strategies Self-Efficacy Scale, *RNLI-C* Chinese version of the Reintegration to Normal Living Index, *ADLs* Activities of Daily Living

## Discussion

We aimed to evaluate the association between participation self-efficacy and participation in community-dwelling mild or moderate stroke survivors. We found that stroke survivors’ participation self-efficacy was significantly associated with their participation, and survivors with higher participation self-efficacy had increased participation. We additionally evaluated the association between stroke survivors’ participation and various demographic and clinical characteristics, ADLs performance, and mobility. Regarding demographic characteristics, participation was significantly associated with educational level, marital status, and pre-morbid financial role in the family while age, sex, and pre-morbid employment did not show significant associations. Regarding clinical characteristics, a history of diabetes or heart disease, use of a walking aid or wheelchair, the presence of haemorrhagic stroke or both ischaemic and haemorrhagic stroke, lesions on both sides, greater frequency of stroke, and higher stroke severity were associated with reduced participation among stroke survivors. We also found that higher levels of mobility and ADLs performance among stroke survivors were significantly associated with increased participation.

While, to our knowledge, this is the first study to specifically explore the relationship between stroke survivors’ self-efficacy in using strategies to manage participation, other studies have shown a correlation between self-efficacy and participation [36]. Stroke survivors with higher self-efficacy levels possess higher coping abilities, commitment, and motivation to set higher self-goals and more effectively achieve their recovery goals, which commonly encompass participation [18]. Participation is associated with survivors’ psychosocial well-being and emotional health [37, 38], including higher community reintegration being correlated with reduced post-stroke depression [39]. Thus, it can be anticipated that rehabilitation interventions aiming to enhance participation self-efficacy may play an important part in facilitating stroke survivors’ holistic recovery beyond the physical domain.

We found that stroke survivors who had a lower educational level, were divorced or widowed, and did not have a pre-morbid financial role in the family were associated with a lower level of participation. A prospective cohort study that followed 390 stroke survivors from hospital admission to 2 years similarly reported poorer social participation in less-educated survivors [40]. With respect to marital status, studies consistently show that it is significantly correlated with social support, which if provided early at discharge in particular is a major predictor of social participation among stroke survivors [41, 42]. Also, our finding of an association between survivors’ financial role and increased participation may be explained by a desire to return to work to continue to earn an income and contribute financially to their family, while the actual terms of their employment may be unimportant [43]. However, in contrast to our findings, younger and female stroke survivors may be more likely to haveincreased participation [40, 44].

With regard to the associations observed between participation and participants’ clinical characteristics, although the existence of comorbidities is generally known to be significantly related to reduced participation, we found that a history of hypertension was not specifically associated with participation [45]. In addition, while assistive aid use in stroke survivors in our study may be linked to reduced participation, previous research has emphasised that the use of walking aids incorporated in daily life may enhance activity and participation in adults with physical disabilities [46]. Aligned with our findings, a 12-month longitudinal study of stroke survivors also highlighted the role of stroke severity, with more severe strokes resulting in much lower participation [47]. Moreover, studies have similarly shown the positive effect of enhanced functional mobility on promoting community reintegration among stroke survivors [48], including improved ADLs and, hence, ADLs performance predicting participation at 1- and 3-months post-stroke [49], whereas survivors with limited ADLs experienced more participation restrictions [40].

Our findings have various possible implications for future stroke rehabilitation programmes. With participation an important aspect of complete stroke recovery, we suggest that it may be worthwhile to consider incorporating participation-focused strategies designed to enhance self-efficacy. In addition, considering the positive association between participation, mobility, and ADLs performance, functional rehabilitation programmes might also address the participation aspect within their activities. Finally, our findings also offer some insights into survivors’ demographic and clinical characteristics that may be associated with adverse participation outcomes, and consequently, pointers to identifying at-risk stroke survivors who may require more attention and support during their recovery.

Our study has several limitations. First, the participants in this study were recruited using convenience sampling, which leads to selection bias and limits the generalisability of our findings. Second, we only aimed to assess associations and a direct causal link between participation self-efficacy and participation cannot be inferred. Third, we excluded stroke survivors with severe cognitive impairment, severe aphasia, or unstable medical conditions, all of whom are known to demonstrate worsened participation outcomes, engagement in social activities, and community reintegration, and commonly in greater need of targeted rehabilitation [44, 50, 51]. Finally, although adjustment was considered for major demographic and clinical characteristics of this population, the association between participation self-efficacy and participation may be confounded by other unassessed factors. Caution is therefore warranted when interpreting our findings.

## Conclusions

Our study shows that participation self-efficacy is significantly associated with participation among Chinese community-dwelling stroke survivors of mild or moderate stroke. As such, rehabilitation programmes may be more effective if they incorporate participation-focused strategies designed to enhance self-efficacy and potentially aid recovery and community reintegration.

## Supplementary Information

**Additional file 1:** Reporting checklist for cross sectional study.

## Data Availability

The datasets generated and/or analysed during the current study are not publicly available due to limitations of ethical approval involving the patient data and anonymity but are available from the corresponding author on reasonable request.

## Abbreviations

MBI: Modified Barthel Index
MBI-C: Chinese version of the Modified Barthel Index
MoCA: Montreal Cognitive Assessment
NIHSS: National Institutes of Health Stroke Scale
PS-SES: Participation Strategies Self-Efficacy Scale
PS-SES-C: Chinese version of the Participation Strategies Self-Efficacy Scale
RMI: Rivermead Mobility Index
RMI-C: Chinese version of the Rivermead Mobility Index
RNLI: Reintegration to Normal Living Index
RNLI-C: Chinese version of the Reintegration to Normal Living Index
ADLs: Activities of Daily Living
